# New York Academy of Medicine

**Published:** 1881-05

**Authors:** 


### NEW YORK ACADEMY OF MEDICINE.

---

*Croupous Pneumonia as an Infectious Disease.*—Dr. Edward Sanders read a paper on the above subject, of which the following is an abstract: With reference to the nature of the disease, there were two theories: first, that it is a local inflammation; and second, that it is an acute infectious disease or essential fever, dependent upon a specific blood-poison. The writer first attacked the older theory that the disease is a local inflammation, and brought the following objections against it: The phlogistic theory does not apply in all cases. Artificial croupous pneumonia cannot be produced in animals by irritating the air-passages. The pneumonia which follows division of the vagus is catarrhal. There is no case upon record which shows that croupous pneumonia has been produced by any of the causes that give rise to other inflammations. Cold has been supposed to be a cause of pneumonia, but there is no foundation for such belief. There is a definite relation between latitude and pneumonia, and Dr. Sanders presented charts illustrating that point. Bronchitis increases as we ascend from the equator, and pneumonia increases the nearer we approach the tropics. Such is true of the United States. Pneumonia is essentially a disease of the winter and spring months. Those following out-door occupations are less liable to the disease than those who have indoor employment, and the disease occurs less frequently in the country than in cities.

With reference to symptoms, a parallelism should exist between the local lesion and the constitutional symptoms, if pneumonia is a local inflammation; whereas the general symptoms of the disease do not bear any relation whatever to the extent of the local lesion, and the treatment now commonly employed is contrary to all accepted notions of local disease. Formerly it was combatted as if it were a local phlegmasia, but the old plans of treatment had been entirely discarded and that now employed does not differ from the treatment commonly adopted for infectious diseases. Local measures are employed, in is true, but they are used solely for the relief of such local manifestations as demand special attention.

How does pneumonia resemble acute infectious diseases? He defined an infectious disease as one dependent upon the introduction into the blood of specific germs capable of reproducing themselves indefinitely. Infection does not necessarily imply contagion; but, under certain circumstances, acute croupous pneumonia possesses a contagious character.

Dr. Sanders then referred to several recorded facts which he regarded as substantiating the belief that pneumonia may be contagious. He next directed attention to the characteristics of infectious diseases, and compared the features of pneumonia with those presented by that class of diseases.

*First.*—Infrequency of occurrence and number attacked. That is, acute infectious diseases do not prevail extensively some years, and when they appear the proportion affected varies very greatly. Pneumonia has occurred only rarely in some years, and it has at times prevailed epidemically in cloisters, prisons, barracks, etc. Special reference was then made to the occurrence of the disease as an epidemic. Again, abortive cases of pneumonia—the occurrence of such cases being acknowledged—indicate their infectious character.

*Second.*—Acute infectious diseases cannot be produced experimentally; neither can croupous pneumonia.

*Third.*—Acute infectious diseases have a stage of incubation. This is not well understood in croupous pneumonia.

*Fourth.*—Acute infectious diseases have a premonitory stage. So does croupous pneumonia.

*Fifth.*—Acute infectious diseases pursue a uniform and classical course. There is no disease which has so uniform and classical a course as lobar (croupous) pneumonia, and local symptoms remain as they do in typhoid fever, etc.

*Sixth.*—In acute infectious diseases there is absence of a direct relation between the constitutional symptoms and the local lesions. So it is with croupous pneumonia.

*Seventh.*—In acute infectious diseases certain complications appear in certain epidemies, and are absent in others. The complications of croupous pneumonia vary in different years.

*Eighth.*—Acute infectious diseases are self-limited. So is croupous pneumonia in a marked degree, and to no other disease with greater propriety can the term self-limited be applied.

*Ninth.*—The rate of mortality varies in each epidemic of an acute infectious disease.

*Tenth.*—In acute infectious diseases there is a localization of morbid changes in some organ or set of organs. In croupous pneumonia the localization is in the pulmonary structures, and consolidation of lung tissue is the essential lesion of the disease.

*Eleventh.*—In acute infectious diseases the use of remedies against the disease itself is useless.

*Twelfth.*—The great characteristic of acute infectious diseases is their specific nature. The producing element for each member of the class is a specific poison acting upon and through the blood.

The main conclusion reached was that croupous pneumonia is an acute infectious disease dependent upon the introduction into the system of a specific poison; that it belongs to the miasmatic-contagious group; and that in all probability the specific poison is taken into the blood by inhalation.

Dr. Austin Flint opened the discussion and said that his views were substantially in accord with those advanced by the author of the paper, and had been placed upon record in a paper which he read before the Medical Society of the State of New York, in 1877, (see *Medical Record*, vol. xii., p. 433), and also in a more recent publication. He was glad to hear Dr. Sanders' remarks with regard to cold as a cause of pneumonia. He thought we should get rid of the old idea that diseases are produced by cold. With regard to this particular disease, he published, about twenty years ago, an analysis of one hundred and forty cases which had escaped the observation of the author of the paper in his researches in the literature of the subject, and the result of that analysis showed, besides other things, that there was nothing whatever to sustain the view that pneumonia is caused by cold.

His statistics also showed a different result from that presented by the author of the paper in another particular, namely, that those whose occupation involves an outdoor life are more likely to have pneumonia than others. Quite possible it was that an analysis of a larger number of cases might show results which would differ from those he had obtained. Dr. Flint then referred to a point not presented in the paper, namely, the treatment of pneumoniac fever, as he had chosen to call it, by antipyretic measures. At Bellevue Hospital three cases had been under his observation, which were treated successfully by the use of the cold pack or the wet sheet, and their average duration was somewhat less than it was ordinarily. The details of the histories of the cases would soon be presented in the form of a paper.

Dr. William H. Draper thought that the onset, the progress and the termination of the lobar pneumonia showed that it is not a fever secondary to a local inflammation; but, on the contrary, that it is an essential fever with a lesion belonging to it. He had been struck with the analogy which it bears to typhus fever and idiopathic erysipelas. Then the question arose, What do we mean by an essential fever? Do we mean nescessarily that it is due to a morbid process excited in the blood by the introduction of a germ? He thought it was very doubtful whether we can give any such definite limitations as that to essential fevers. He doubted if any one believed that rheumatism depends upon the introduction into the blood of any infectious poison, and yet it must be regarded as a fever. He thought that the author of the paper had perhaps fallen into an error in restricting the definition of infectious disease in that way.

Dr. Draper was not prepared to say that croupous pneumonia always depended upon the same cause. He was not prepared to admit that the epidemics of pueumonia which had been alluded to in the paper were made up of cases of the very same disease, which, occurring sporadically we call croupous pneumonia, no more than he was willing to admit that every case of cerebro-spinal miningitis is a case of epidemic cerebro-spinal meningitis. The epidemics of pneumonia mentioned, like those of cerebro-spinal miningitis, occurred under conditions which can only suggest the operation of some infecting cause. He was not prepared to say that the cases of croupous pneumonia which we see now in hospitals and in private practice are the same disease as that which occurs under circumstances when pneumonia becomes epidemic. It seemed to him that the question of essential fevers embraced something in explanation of them besides the introduction of germs. He believed that, under the influence of exhausted nerve-power, a lesion may occur which cannot be distinguished from the lesion of croupous pneumonia. He regarded it as a striking fact in the history of pneumonia that a large majority of cases occur among men, and among those who are exposed to the vicissitudes of the weather in severe occuaptions, and all those circumstances which tax the nervous system in a peculiar manner. He also thought that it might be a mistake to say that the lesion of croupous pneumonia can only be produced by the introduction of a specific poison into the blood by inhalation.

Dr. A. L. Loomis thought that Dr. Draper had struck the key-note of the discussion, that is, are we prepared to accept the doctrine that pneumonia depends upon a specific poison which produces a constitutional disease with a local lesion? Some five years ago, in a discussion on pneumonia held in a sister society, he stated that he did not believe pneumonia to be a local disease. Since that time experience has so convinced him that he no longer, under any circumstances, regards croupous pneumonia as a local affection. Nor was he prepared to regard it as a specific constitutional disease with a local lesion. In other words, he was not prepared to accept the doctrine that in order to have pneumonia one must receive the specific pneumonia poison, and that that poison alone has the power to develop the local changes which characterize the disease. He preferred to occupy a middle ground—that pneumonia, like many other inflammations, may be developed by a variety of poisons. A man loaded with alcohol is especially liable to pneumonia. A man weighted down with the specific poison of malaria, if it is specific, when brought under certain influences which depress the nervous system, is very liable to get pneumonia. The same was true with regard to insufficient action of the kidneys, permitting the system to become poisoned with urea, and in all these cases there is the same record, so far as pathological changes are concerned, but the disease depends upon different causes. They are infectious pneumonias, but the infection is not the same in each case; and it seemed to him that this is exactly the point upon which the discussion hinges.

As regards the action of cold in the development of pneumonia, there could be no question that it is not the essential cause; but it probably is an exciting cause in many cases. If the relationship of pneumonia to acknowledged acute infectious diseases was carefully examined, it would be found that all hangs upon nervous phenomena. It is the chill, or nervous shock, which marks its beginning; and, usually, a nervous shock indicates the commencement of most infectious diseases. The patient does not die because the lungs are crowded, but because there is failure in nervous supply; heart-failure, it may be called, but the heart-failure comes through the effect upon the nervous system.

The Academy then adjourned.

---

### OBSTETRIC SECTION OF THE NEW YORK ACADEMY OF MEDICINE.

*The Treatment of Pleurisy in Children.*—Dr. J. Lewis Smith read a paper on the above subject, in which he spoke of the treatment appropriate to each of the three stages:

1. The stage which precedes the effusion;
2. The stage of effusion; and
3. The stage of absorption and convalescence.

In the beginning of the disease measures should be adopted which are appropriate for reducing inflammation and reducing exudation. The abstraction of blood in idiopathic pleurisy may be beneficial if judiciously employed, but only one or two or three leeches should be employed in a robust child two, three or four years old. As a rule, the loss of blood is injurious in all cases of secondary pleurisy, such as follow scarlet fever, etc., and also if the quantity of effusion is great. Emollient and simply irritating poultices are serviceable in the first stage, and he recommends a mixture of *one* part of mustard to *sixteen* of linseed. It should be made very wet, spread thin, applied over the chest in front and behind, covered with oil-silk, and changed twice in twenty-four hours. For children under six or seven months of age, rubbing the chest with camphorated oil, and applying a simple poultice may be sufficient.

Blistering at this early stage of the disease should not be employed, as it increases the inflammation, and Dr. Smith has seen a case which terminated fatally, in which there was found an increased area of inflammation corresponding exactly in situation, size and shape to a blister that had been applied.

The indications for the use of internal remedies in the first stage are to diminish the frequency of the pulse, relieve the pain, and allay the cough.

To a child *three* years old the tincture of aconite may be given in dozes of half a drop, and for a child *six* years old in doses of *one* drop, every three hours for two or three days. In the first stage of primary pleurisy the cardiac sedatives may be used; but digitalis is a safer and better remedy in all other cases, and it can also be used in the second stage.

To a child *two* years old the tincture of digitalis may be given in doses of *one drop every three hours*, and to a child five years old *two* drops with the same interval. An opiate is ordinarily required: Dover's powder, one to three grains, every three hours. Hyoscyamus may be used to relieve the pain and cough; digitalis may be combined with an opiate; and morphine and aconite may be combined.

In secondary pleurisy digitalis is preferable to aconite.

In the *second* stage, unless the effusion is small, measures designed to remove it are required. The propriety of using blisters in this stage is very doubtful.

A relaxed condition of the bowels favors absorption of serous effusion. Diaphoretics do not aid much in the removal of the fluid. Pilocarpine produces a depressing effect, which renders it unsafe.

Diuretics and tonics are beneficial. Digitalis, with the acetate of potash, is very serviceable.

| | | |
|---|---|---|
| ℞. Infus digitalis | - - - - | ℥ iv. |
| Potass. acetat | - - - - - | ʒ j. |

M. S.—Teaspoonful every three hours, to a child four or five years old.

Bitter tonics are especially useful in this stage, and the acetate of potash may be combined with a decoction of cinchona, with good results. A full amount of nutriment should be taken with but little fluid. Of course, the suggestion to use a dry diet and diminish the quantity of drink is not applicable to young children. If the appetite and general health are good, and there are no symptoms due to the presence of the fluid, but little medication is necessary. If there are such symptoms and the fluid does disappear, the question of surgical interference arises, and the indications for it are the following:

*First.*—Oppressed breathing due to the liquid present, whether it be sero-fibrinous, purulent or hemorrhagic.

*Second.*—If there be flat percussion-note over the entire affected side, with displacement of the heart, even if there be no dyspnœa, for the latter may occur suddenly.

*Third.*—Moderate effusion, without material decrease in quantity by absorption after some weeks of treatment. There is danger that catarrhal pneumonia terminating in cheesy pneumonia and tuberculosis may occur in portions of the compressed lung. Besides, the longer the lung is compressed, the slower will it return to normal expansion after the pressure has been removed.

*Fourth.*—A moderate quantity of fluid co existing with disease of the opposite lung, or of the lung of the affected side.

*Fifth.*—Extension of the inflammation to the pericardium. Pericarditis as an extension of the inflammation is not infrequent.

*Sixth.*—The extension of valvular lesion of the heart.

*Seventh.*—The presence of pus; empyema.

The operation of thoracentesis should be performed in the eighth intercostal space, on a line perpendicular with the angle of the scapula. The admission of air to the pleural cavity should be carefully avoided. The thickness of the thoracic wall is about half an inch; in emaciated children it is less. Introduction of the canula to the depth of *one inch* is sufficient to pass the exudation and allow the liquid to pass through the canula. The sharp needle should not be used. Washing out the pleural cavity is unnecessary; it is injurious rather than beneficial, except in cases in which the pus is offensive. To empty the pleural cavity and approximate pleural surfaces is the indication. Dr. Smith thinks there will be a reaction against the removal of a portion of the ribs in cases of empyema.

Dr. A. C. Post favored free incision rather than aspiration in empyema.

Dr. Caro disapproved of bloodletting, even in the first stage of pleurisy. He uses bromide of potassium in doses of two or three grains every one or two hours, for the purpose of reducing the capillary congestion. Aconite may be used if there is much elevation of temperature. He especially favored the use of acetate of potassium in free doses in the second stage.

Large doses of calomel also diminished capillary congestion and favored resolution of the inflammation. Externally, in the second stage, he recommended the tincture of iodine or Lugol's solution, covered with oil silk. He favored the internal use of jaborandi in infusion, and believed it to be preferable to hypodermic injections of pilocarpin. Take *two* drachms of the leaves to three or four ounces of water, and give the whole in two or three doses.

The Section then adjourned.

